# Combined Use of *Lachancea thermotolerans* and *Schizosaccharomyces pombe* in Winemaking: A Review

**DOI:** 10.3390/microorganisms8050655

**Published:** 2020-04-30

**Authors:** Santiago Benito

**Affiliations:** Department of Chemistry and Food Technology, Polytechnic University of Madrid, Ciudad Universitaria S/N, 28040 Madrid, Spain; santiago.benito@upm.es; Tel.: +34-913-363-710 or +34-913-363-984

**Keywords:** *Lachancea thermotolerans*, *Schizosaccharomyces pombe*, malic acid, lactic acid, aroma, color, wine

## Abstract

The combined use of *Lachancea thermotolerans* and *Schizosaccharomyces pombe* is a new winemaking biotechnology that aims to solve some modern industrial oenology problems related to warm viticulture regions. These areas are characterized for producing musts with high levels of sugar that can potentially be converted into wines with elevated ethanol contents, which are usually associated with high pH levels. This biotechnology was reported for the first time in 2015, and since then, several scientific articles have been published regarding this topic. These reported scientific studies follow an evolution similar to that performed in the past for *Saccharomyces cerevisiae* and *Oenococcus oeni*; they start by reporting results for basic winemaking parameters at the beginning, later continuing with more advanced parameters. This review compares the results of different researchers that have applied this new biotechnology and have studied wine quality parameters such as ethanol, glycerol, malic acid, lactic acid, amino acids, aroma compounds, or anthocyanins. It is shown that the new biotechnology is repeatedly reported to solve specific winemaking problems such as the lack of acidity, biogenic amines, ethyl carbamate, or undesirable color losses. Such results highlight this biotechnology as a promising option for warm viticulture areas.

## 1. Introduction

The use of non-*Saccharomyces* is becoming popular in modern winemaking after a prolonged domination of *Saccharomyces cerevisiae* at the industrial level that started with the commercialization of dry yeast in the past. The search for new products different from standardized ones has moved companies and researchers to focus on new yeast species [1,2], different from classical *S. cerevisiae* and able to add differentiation in wine quality parameters. The main studied species are *Torulaspora delbrueckii* [3], *Lachancea thermotolerans* [4,5], *Metschnikowia pulcherrima* [6], *Schizosaccharomyces pombe* [7], *Pichia kluyveri* [8], *Hanseniaspora* spp. [9], and *Starmerella bacillaris* [10].

**Figure 1:**
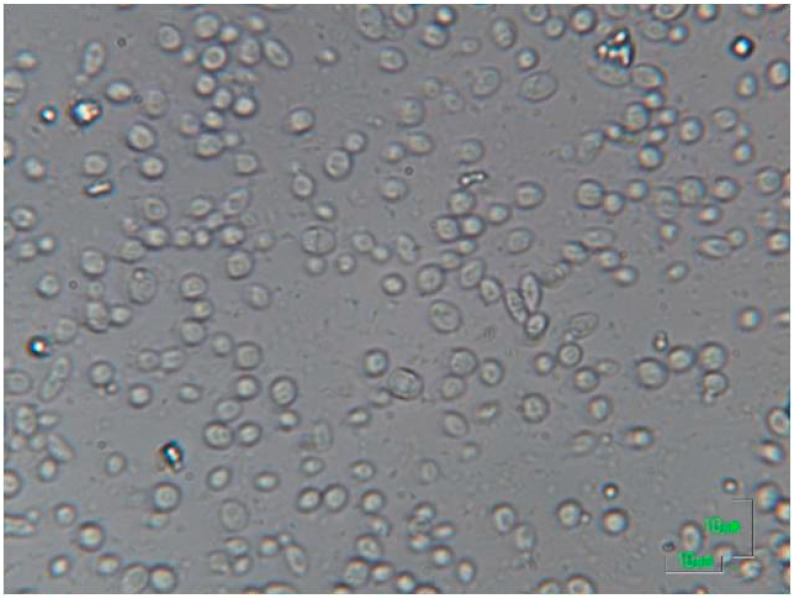
Microscopic observation of an alcoholic fermentation ending in a low acidic must/wine with high content in sugar. *Saccharomyces cerevisiae* are the cells between 5 and 10 µm, while *Oenococcus oeni* are the small cells of about 1 µm. In this difficult alcoholic fermentation ending, *S. cerevisiae* cells are still difficultly fermenting sugar in an already high alcohol concentration of about 15 % *v*/*v* after 20 days of fermentation. A high population of lactic bacteria (*Oenococcus oeni*) start to be evident although the alcoholic fermentation is not properly finished yet.

*Lachancea thermotolerans* (LT) is able to increase wine acidity in warm viticulture areas whose grapes commonly suffer from a lack of acidity related to fruit ripening under hot conditions, where most acids are combusted [11]. The acidification process takes place through the production of lactic acid during alcoholic fermentation (AF) [5,12,13,14,15,16,17]. Additional improvements reported for *L. thermotolerans* fermentation include better biocontrol [18], a low volatile acidity production [4], a greater aroma complexity [19], and an increase in color intensity [20]. Nevertheless, the main disadvantage of using *L. thermotolerans* at the industry scale is its limited fermentation power that makes it impossible to ferment wines with ethanol concentrations over 10% without combining it with a more powerful species fermenter [4,5,19]. Although *Schizosaccharomyces pombe* (SP) was originally applied to de-acidify high-acidic wines from northern Europe with high contents of malic acid (greater than 6 g/L) that make them extremely sharp for regular consumers [21,22], it has been recently used in warmer areas in order to avoid the need to perform malolactic fermentation (MLF) in difficult scenarios such as low-acidic and extremely sweet wines [7]. Under these circumstances, lactic acid bacteria (LB) may metabolize fermentable sugar into acetic acid during AF, ultimately seriously deteriorating the quality of the wine (Figure 1). Although the main oenological ability of *S. pombe* is the conversion of malic acid into small amounts of ethanol, improving wine microbiological stabilization before bottling, other interesting improvements such as the release of polysaccharides [23,24], better color [25], or a reduction in biogenic amines and ethyl carbamate have also been reported [7]. Several authors have reported a high production of acetic acid as the main undesirable collateral effect regarding the use of *S. pombe* in alcoholic beverage industries [22,26,27,28,29]. Table 1 summarizes the main advantages and disadvantages of *L. thermotolerans* and *S. pombe* yeast species applied to winemaking.

Traditionally, the achievement of stable wines from a microbiological point of view is based on alcoholic fermentation (AF) performed by *Saccharomyces cerevisiae* (SC), and later, malolactic fermentation (MLF) performed by *Oenococcus oeni* (OE). This classical methodology (SC+MLF) remains the most appropriate for temperate climates. Nevertheless, modern winemaking faces new problems such as those produced by climate change in warm areas [11]. The main problem in these warm areas is the high content of fermentable sugar (more than 255 g/L), which makes it difficult to properly complete the transformation of glucose and fructose into ethanol by *S. cerevisiae* from a technological point of view (Figure 1). Additionally, modern consumers demand wines without such a high ethanol content, preferring instead to enjoy other wine virtues such as its aroma or body. The other main problem is the lack of acidity in very ripe grapes. This lack of acidity unbalances the flavor of wine and generates technical problems related to high pH. Under high pH, secondary fermentation by lactic bacteria (MLF) may result in high levels of volatile acidity, biogenic amines, and ethyl carbamate, resulting in not only the problem of sensory quality but also affecting food safety and human health.

**Figure 2:**
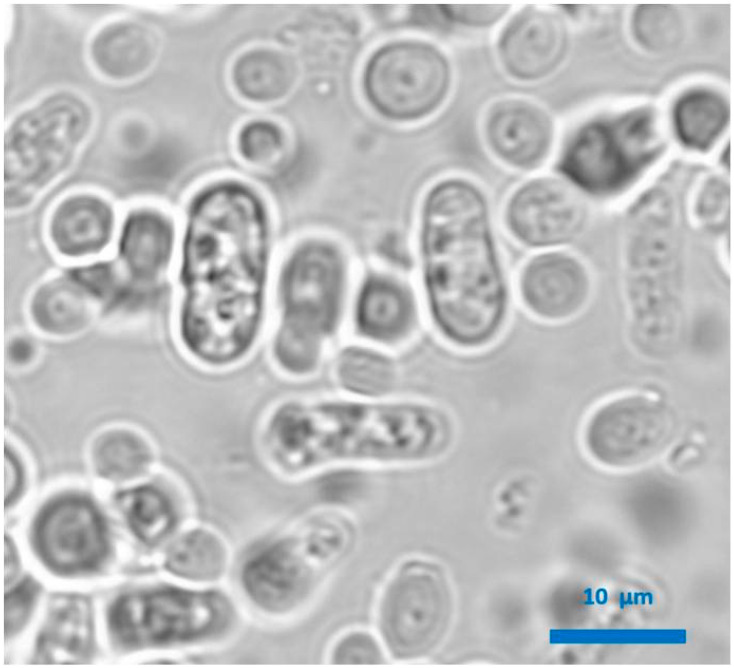
Microscopic observation of a wine produced with combined *Lachancea thermotolerans* and *Schizosaccharomyces pombe* alcoholic fermentation. *S. pombe* cells are rectangular, while *L. thermotolerans* cells are spherical.

**Figure 3:**
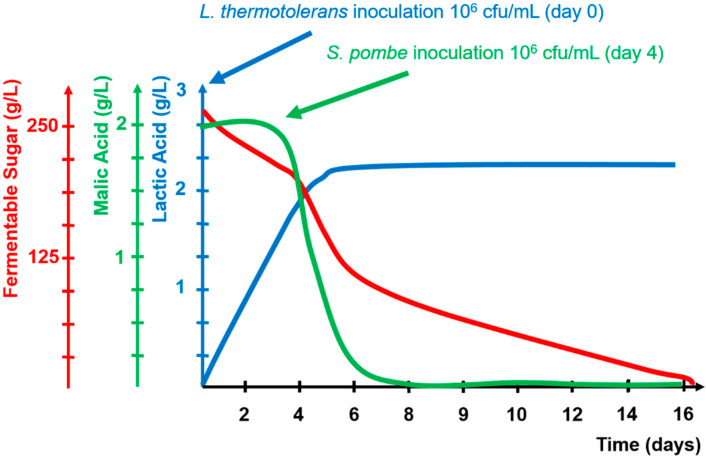
Kinetics of fermentable sugars, lactic acid, and malic acid during alcoholic fermentation combining *Lacchancea thermotolerans* and *Schizosaccharomyces pombe*.

Under this new climate change scenario, new biotechnologies to improve the necessary industrial malic de-acidification for microbial stability have been developed with the purpose of adapting them to the industrial problems of warm viticulture areas. One new biotechnology is based on the use of lactic bacterial species different from the classical *Oenococcus oeni,* such as *Lactobacillus plantarum,* which is able to consume malic acid during the first stages of AF without consuming any fermentable sugars. Another new approach is the combined use of *Lachancea thermotolerans* and *Schizosaccharomyces pombe* (LT+SP) during AF (Figure 2). In this combination, when applied to warm viticulture areas, *S. pombe* consumes all of the malic acid, converting it into small amounts of ethanol and carbon dioxide. Thus improving the desired industrial microbiological stabilization based in total sugar and malic acid consumption that reduces the risk of re-fermentation by LB due to the lack of nutrients. In addition, *L. thermotolerans* generates lactic acid that compensates for the lack of acidity produced by the disappearance of malic acid and generates natural wines of optimal acidity from low-acidic grape juices. Figure 3 shows the evolution of some parameters during LT+SP AF.

The biotechnology that combines the use of *L. thermotolerans* and *S. pombe* (LT+SP) was performed for the first time in 2015 [30,31], and basic wine quality parameters such as malic acid, lactic acid, pH, glycerol, and color intensity were studied. The ensuing studies started to research more advanced parameters such as wine volatile compounds [32], amino acids [32], biogenic amines [30,32], anthocyanins [33,34,35], or polysaccharides [36]. Studies regarding the new biotechnology started following a similar evolution as that which took place in the past for the classical methodology (SC+MLF) based on AF by *S. cerevisiae* and MLF by *O. oeni*. Additionally, several different researchers have utilized the new methodology [34,37,38,39] during recent years, making possible a comparison between the results of different studies. The present study aimed to compare all of the studies regarding LT+SP combined biotechnology, the number of which has increased, as well as to try to explain the observed discrepancies and to establish conclusions. Table 2 summarizes the main comparison points between the different LT+SP studies performed until now. Additionally, it is hoped that this review will encourage other researchers to perform similar trials to compare the latest results of this new biotechnology (LT+SP), to avoid possible design mistakes, and to add new, unpublished results.

## 2. The Influence of *L. thermotolerans* and *S. pombe* on the Main Wine Quality Parameters

### 2.1. Fermentation Kinetics

Most studies report longer fermentation times for fermentation combining LT and SP than the controls where AF was performed only by *S. cerevisiae*. The reported delays vary from 4 to 8 days [30,32,33]. Other studies report no significant differences in AF fermentation time [34,38,39]. Nevertheless, the studies that include a control that performed classical MLF report longer periods for SC+MLF, where most of the time is consumed during the MLF performed by LB to improve microbiological stabilization. The time of the MLF varies from 21 to 33 additional days [30,32,33,36]. According to these results, LT+SP biotechnology is between 17 to 25 days faster than the classical method (SC+MLF). We must also take into account that in these studies, LB were inoculated at a suitable concentration and performed the deacidification stabilization at a favorable controlled temperature of 18 °C. Spontaneous MLF fermentation without optimal conditions could notably delay the final wine microbial stabilization. Most studies show that when *S. pombe* is inoculated, *L. thermotolerans* cells disappear rapidly to undetectable levels in about 4 [30], through 5 [32,33], to 6 [39] days. One interesting option to reduce the total fermentation time is *S. cerevisiae* and *O. oeni* co-inoculation, nevertheless that methodology in wines with high pH and sugar content may deviate in difficult alcoholic fermentation endings as that showed in Figure 1.

### 2.2. Ethanol

*L. thermotolerans* is able to ferment ethanol concentrations of up to 9–10.5% *v*/*v* [5,19]. These ethanol concentrations can be suitable for beer or sparkling-based wines, but not for regular dry red wines. Contrarily, the *Schizosaccharomyces* genus is able to ferment concentrations of up to 15% *v*/*v* during winemaking, depending on the strain [7], and even up to 20% *v*/*v* with magnesium nutrient supplementation [40]. Regarding *S. pombe* and *L. thermotolerans* symbiosis, *S. pombe* is the species responsible for proper AF ending in regular dry wines due to its higher fermentative power.

*L. thermotolerans* is reported to produce lower final ethanol levels in combined fermentation with *S. cerevisiae,* i.e., concentrations of about 0.2–0.5% *v*/*v* [19,20]. This phenomenon is explained by the metabolism of some yeast species, which allows the generation of higher concentrations of metabolites different from ethanol, such as yeast biomass, glycerol, or pyruvic acid, that decrease the amount of carbon atoms metabolized into ethanol [2,31]. *S. pombe* is occasionally described as a slightly lower ethanol producer than *S. cerevisiae*, i.e., concentrations of about 0.2% *v/v,* due to its highly developed glycerol–pyruvic pathway [7] or polysaccharide formation [23,36]. Nevertheless, when *S. pombe* ferments juices with a high initial content of malic acid, the final ethanol concentrations notably increase by more than 1% *v*/*v* [27]; malic–ethanol fermentation is able to mask any other process able to reduce the final ethanol concentration.

The first study regarding the combined use of LT and SP reported a reduction of about 0.52% *v*/*v* between the classical fermentation SC+MLF and LT+SP [30]. Later works reported similar ethanol reductions of 0.5% *v*/*v* [32], 0.41% *v*/*v* [33], and 0.4–0.5% *v*/*v* [34], or higher ones of 0.71–1.27% *v*/*v* [37] and 1.27–3% *v*/*v* [39]. Every case reported ethanol reductions, although in notably different amounts.

The LT+SP biotechnology is reported to be suitable for fermenting grape juices with a probable alcohol degree of up to 14.88% (245 g/L of total sugar), fermenting all fermentable sugars to dryness [32]. This result indicates that LT+SP biotechnology is of great interest for warm viticulture areas where sugar contents are high and the content of malic acid and the total acidity are low [2,11]. Although most works report moderate ethanol reductions from 0.3% to 0.5% *v*/*v*, the observed ethanol reductions greater than 1% *v*/*v* up to 1.27% or 3% *v*/*v* [37,39] reported by some authors are very promising. This ethanol decrease could be used to reduce the potential high ethanol content of wines from warm viticulture areas.

The slight reductions in final ethanol concentrations of about 0.2–0.5% *v*/*v* [30,32,33] for combined LT+SP fermentations could be explained by *L. thermotolerans* metabolism and the performance of *S. pombe* in juices with a low content of malic acid. Higher reductions exceeding 1% *v*/*v* [37,39] could be produced as a result of the interactions between both species. Both studies [37,39] reported that pure *S. pombe* fermentation (SP) and *L. thermotolerans* and *S. cerevisiae* combined fermentation (LT+SC) had lower ethanol reductions than the LT+SP combined option. Therefore, this phenomenon must be more deeply studied.

### 2.3. Glycerol

Glycerol is the third main compound in wine after water and ethanol. High levels are commonly related to better sensory properties such as structure, body, or sweetness. Some works reported that *L. thermotolerans* [12,16] and *Schizosaccharomyces* [23] were higher glycerol producers than the *S. cerevisiae* controls, i.e., increases of 0.5 to 1.5 g/L. The first study regarding fermentation combining *L. thermotolerans* and *S. pombe* reported a higher glycerol concentration for LT+SP fermentation, i.e., 0.69 g/L [30], than for the SC control. Later studies also reported higher levels of 0.46 g/L [32], 0.71 g/L [33], or 0.27 g/L [36]. These differences may be explained by the strain variability for this parameter, reported for species from the *Schizosaccharomyces* genus to reach 21%, similar to that reported for the *Saccharomyces* genus [7,22,23]. Nevertheless, interactions between species and other parameters must be studied in-depth. Some studies reported higher glycerol production in pure control fermentations of *S. pombe* and *L. thermotolerans* separately than for the *S. cerevisiae* control, namely, 1.51–2.93 and 1.85 g/L, respectively, while combined LT+SP fermentations produced between 0.64 and 2.4 g/L less glycerol than the *S. cerevisiae* control [37].

### 2.4. L-Malic Acid

*L. thermotolerans* is described as a moderate malic acid consumer, with consumption ranging from 10% to 25% [12,14,19]. Some authors reported higher consumptions, up to 50%, for grape juices with very high contents of malic acid [20,41]. The *Schizosaccharomyces* genus is described as the highest malic acid consumer among yeast, being able to consume up to 100% of total initial malic acid [7]. This phenomenon takes place due to the malo-ethanolic fermentation that converts one molecule of malic acid into one molecule of ethanol and two molecules of carbon dioxide. For that reason, *S. pombe* is responsible for the total consumption of malic acid in combined LT+SP fermentations. The total consumption of malic acid allows the generation of microbially stable wines without the use of LB. This fact is of great interest in the production of wines in warm viticulture areas, where it is very difficult to perform classical MLF due to the high ethanol and sugar contents. In this scenario the risk of AF and MLF taking place simultaneously during difficult AF endings increases (Figure 1). Additionally, the high pH levels in warm viticulture regions are responsible for undesirable MLF deviations, such as high levels of acetic acid, biogenic amines, and ethyl carbamate [42].

All studies report combined LT+SP fermentation to consume most of the malic acid down to levels between 0 to 0.2 g/L. These values improve wine stability from a microbiological point of view, which must be achieved before the bottling of red wine in order to avoid possible re-fermentation by LB. The first study reported a reduction in malic acid of 99.9% [30], and other studies reported similar results of 100% [32,33,36], 95–100% [37], and 72–75% [34]. The studies that included a classical malolactic fermentation control (SC+MLF) performed by LB did not show statistical differences in the final malic acid concentration when compared to the LT+SP trial [30,32,33,36]. To include a control that performs classical MLF is crucial in the study of this biotechnology, as it is not possible to establish proper conclusions through comparison with a simple *S. cerevisiae* (SC) control that does not perform MLF. These types of wine are not made in real industry as they are not stable, from a microbiological point of view, to be commercialized in the market. As only half of the studies included these controls, the design of the experiments must be revised for future studies, involving malic de-acidification stabilization strategies to establish proper result comparisons and conclusions.

All studies that report the kinetics of malic acid degradation [30,32] report it to start after 24 h of *S. pombe* inoculation, and the total malic acid degradation length varies from 48 to 72 h. This fact makes it also possible to easily inoculate a regular *S. cerevisiae* strain, once the consumption of malic acid is detected and a great amount of fermentable sugars still remain in the media.

### 2.5. L-Lactic Acid

Lack of acidity in grape juice is one of the main problems in warm viticulture areas. The main option to acidify grape juice is the addition of commercial tartaric acid, citric acid, or, recently, lactic acid. The main advantage of lactic acid is that it is stable, while tartaric acid is unstable from a chemical point of view (it may precipitate as it combines with potassium), and citric acid is unstable from a microbiological point of view (lactic bacteria metabolize it into acetic acid). Classical MLF converts each malic acid gram into 0.67 g of lactic acid and 0.33 g of carbon dioxide, achieving the desired microbiological stabilization but increasing pH by about 0.1 units per metabolized gram of malic acid. The use of *S. pombe*, even in small proportions in initial starters, allowing winemakers to easily improve the desired microbial stability in warm viticulture areas, avoiding possible collateral effects that could appear during difficult MLF at high pH. However, these wines are characterized by a strong lack of acidity, as malic acid is converted directly into ethanol without the production of lactic acid, and the increase in pH is usually several times higher than that in regular MLF. The reason is that these wines are usually acidified with high amounts of commercial acids. Nevertheless, the use of commercial acids is regulated by legal limits of 2 g/L of tartaric acid and 1 g/L of lactic acid in Europe, and high acidification notably increases the final price of wine.

*L. thermotolerans* has become the most popular non-*Saccharomyces* species in warm viticulture areas [4,5,19] due to its ability to naturally increase acidity. *L. thermotolerans* naturally produces lactic acid [12,13,14,43] during AF from sugar metabolism [44,45] without consuming malic acid or citric acid and consequently without increasing acetic acid [4]. *L. thermotolerans* is able to naturally produce up to 6 g/L of lactic acid depending on the initial inoculum and the time of performance [19]. This value is much higher than the allowed limit of artificial lactic acid addition in Europe. Additionally, the final economical cost is much cheaper than the equivalent addition of synthetized commercial food-quality chemical products. *L. thermotolerans* combined with LB, such as *Oenococcus oeni,* increases the final value of lactic acid by about 2.73 g/L [30,46], reducing the pH by about 0.2 units compared to the regular MLF control.

All studies regarding combined LT+SP use that included an appropriate control to perform classical MLF reported higher levels of the final lactic acid concentration, i.e., 2.42 g/L [30], 2.68 g/L [32], 1.56 g/L [33], and 0.5 g/L [36] higher than the MLF control. The differences would be even higher if the results were compared to a control that only performed AF (SC). Studies that did not include an MLF control reported increases in the lactic acid levels of combined LT+SP of 0.59–0.71 g/L [37], 0.3–0.4 g/L [34], and 2.4 g/L [39]. These results show that fermentation involving *L. thermotolerans* always results in higher final lactic concentrations than conventional MLF.

All studies that monitored lactic acid production in LT+SP fermentations [30,32] reported most lactic acid to be produced during the first stages of AF when *L. thermotolerans* is highly active. The production starts to slow down when ethanol reaches 4–6% and other competitor yeast species better adapted to ethanol such as *S. pombe* or *S. cerevisiae* are inoculated. Depending on the delay of *S. pombe* inoculation the production of L-lactic acid can be regulated.

### 2.6. Citric Acid

Fermentations involving combined LT+SP use showed identical final concentrations of citric acid than the original grape juice. However, most studies that included a control to perform MLF reported significant decreases in citric acid of about 90% during the second fermentation developed by LB [30,32,33,36]. This undesirable phenomenon is commonly observed during MLF processes that take place at high pH levels, such as those usually developed in warm viticulture areas [42,47]. Under these circumstances, citric acid is metabolized into acetic acid by LB, increasing the final volatile acidity concentration and reducing the quality of the wine.

### 2.7. Acetic Acid

Acetic acid is a volatile acid, responsible for the vinegar character in wines when it appears in concentrations greater than 0.8 g/L [48]. *L. thermotolerans* is traditionally described as a lower producer of acetic acid than *S. cerevisiae,* with concentrations varying from 0.1 to 0.3 g/L [14,15,43]. However, *S. pombe* is traditionally described as a higher producer of acetic acid compared to *S. cerevisiae* [7]. Several works report pure *Schizosaccharomyces* pombe fermentations (SP) to produce final acetic acid concentrations over the default threshold of 0.8 g/L [26,27,38,47]. Nevertheless, during recent years, several strategies have been developed to reduce this disadvantage. The first methodology developed to reduce the final volatile acidity problem is that of fermentation combining *S. pombe* and *S. cerevisiae* [7]. The use of immobilized *S. pombe* cells allow to easily remove them before the acetic acid reaches high concentrations [7]. A newer technology is *Schizosaccharomyces* strain selection [21,22,49], which allows winemakers to select strains able to produce moderate-to-low acetic acid levels; such studies report a strain variability from 0.3 to 1.2 g/L with respect to acetic acid production. The last option is to perform fed-batch fermentation, which allows *S. pombe* to produce wines with no detectable levels of acetic acid [28,29]. Using this technology, some studies reported final concentrations of acetic acid less than 0.2 g/L in wines fermented by *S. pombe* [41].

The studies regarding combined LT+SP fermentation reported no statistical differences between LT+SP and the pure *S. cerevisiae* fermentation control [30,32,33,36]. Other studies reported lower concentrations of 0.4 g/L [34] or those varying from 0.16 to 0.21 g/L depending on the combined modality [39]. However, one study reported higher concentrations for combined LT+SP fermentation, varying from 0.21 to 0.51 g/L [37], compared to the pure *S. cerevisiae* (SC) control without performing MLF. All of the studies that included a control trial that performed MLF reported a statistical increase in the acetic acid concentration of 0.08 [30], 0.11 [32], 0.12 [33], or 0.11 g/L [36] after this biological process. These phenomena could be related to the citric acid metabolism by LB, reported to increase acetic acid during MLF [30,47]. According to these results, most studies reported lower final levels of acetic acid for LT+SP and always lower than the controls that performed MLF.

### 2.8. pH and Total Acidity

The changes produced in lactic acid by *L. thermotolerans* [50] and in malic acid by *S. pombe* [51] directly influence the pH and total acidity of the final wines. Some studies reported combined LT+SP fermentation to produce wines with a lower pH than the *S. cerevisiae* control, by about 0.11 [30], 0.21 [32], 0.18 [33], or 0.45–0.49 [39]. However, other studies reported pH increases of 0.3–0.35 [37] or 0.1–0.2 [34]. These differences are explained mainly by the production of lactic acid by *L. thermotolerans,* which varies from 0.59 to 3.41 g/L depending on the modality of the combined fermentation. Sequential fermentations always result in lower pH due to the longer duration of *L. thermotolerans,* which produces higher levels of lactic acid. Combined initial co-inoculations usually achieve more moderated contents of lactic acid due to the competition with other species. Under these circumstances, malic acid de-acidification by *S. pombe* has a stronger influence on the final pH.

### 2.9. Acetaldehyde

*S. pombe* usually produces higher levels of acetaldehyde than *S. cerevisiae* controls, by about 25–40%, depending on the selected strain, but levels are less than the faulty threshold when the strains are properly selected [7,22]. Acetaldehyde is described to exert a positive effect on color stabilization through the formation of combined colored stable forms such as vitisin B [33]. This effect is considered positive when the concentration is below the faulty threshold of 125 mg/L [7]; above this concentration, wines may show oxidation aroma notes [52]. Accordingly, combined LT+SP fermentation results in higher levels than pure *S. cerevisiae* fermentation without MLF, of 13% [33] or 25% [36], but lower than the pure *S. pombe* control, by 16% [33] or 14% [36]. Studies that include controls that perform MLF reported significant statistical decreases in acetaldehyde to final levels less than 2 mg/L with reductions of 97% and 95% compared to the LT+SP control. The maximum reported levels of acetaldehyde for LT+SP fermentations are 46 and 64 mg/L, which are below the faulty threshold of 125 mg/L.

### 2.10. Amino Acids

Although there are previous studies regarding the amino acid profiles of wines fermented by *L. thermotolerans* [15,50] and *S. pombe* [22] in pure fermentations, there is only one study that reports the final amino acid profile of a wine produced by a combined LT+SP fermentation [32]. Therefore, further studies need to be performed before establishing general conclusions regarding the new biotechnology and its influence on the amino acids in wine. Some amino acids can be metabolized by yeast into higher alcohols; these aromatic alcohols can mask other desirable aromas when their concentration is over 500 mg/L [48]. The abovementioned study reported 29%, 60%, and 55% higher concentrations of amino acid precursors of higher alcohols, such as threonine, isoleucine, and leucine, respectively, compared to *S. cerevisiae* and *O. oeni* controls (SC; SC+MLF). These results were related to lower levels of the higher alcohols 1-propanol, 2-methylbutanol, and 3-methylbutanol. This phenomenon was previously observed in other non-*Saccharomyces* such as *Torulaspora delbrueckii* [3] and *Metschnikowia pulcherrima* [6,8]. LT+SP fermentation showed no statistical differences in histidine compared to the pure *S. cerevisiae* fermentation, while MLF had statistically lower concentrations, by about 12 mg/L. Histidine is the precursor of histamine and it is usually metabolized by LB during MLF into the hazardous biogenic amine. Nowadays, histamine constitutes the main problem of food safety in wines [51]. Additionally, we have to take into account that wines fermented through LT+SP are stable from a microbiological point of view and do not contain malic acid, residual sugars, or other nutrients that could be used by LB. LT+SP results in lower concentrations of glycine than SC fermentation and SC+MLF, by about 40%. SC+MLF results in lower levels of arginine than LT+SP and SC fermentations, by about 12%, as well as lower levels of alanine, by about 15%.

### 2.11. Polysaccharides

Polysaccharides improve wine quality in parameters related to the sense of taste in the mouth, usually described as body, roundness, or low astringency [36,52]. The polysaccharides mannoproteins are released into the media from yeasts during AF or aging over lees processes. These polysaccharides are the second most abundant group, after grape polysaccharides denominated as arabinogalactan-proteins. *Schizosaccharomyces* is reported as the yeast genus with a sixfold higher release of polysaccharides than *S. cerevisiae* strains selected for this purpose [23]. *L. thermotolerans* is described as having a moderate polysaccharide release, producing similar values to those obtained for *S. cerevisiae* [53]. The first research that studied the topic of combined LT+SP fermentations used an indirect indicator to estimate the content of polysaccharides in wine; this index was termed the ethanol index. This study reported that LT+SP fermentation had a higher ethanol index than SC+MLF fermentation, by about 18%, indicating that the new biotechnology produced higher levels of polysaccharides [32].

A later study quantified the released mannoproteins through a study of hydrolyzed mannose [36]. LT+SP fermentations resulted in lower levels of mannoproteins than obtained under pure fermentation by *S. pombe* (SP), but twice as high as the *S. cerevisiae* (SC) control. The chemical parameter positively influenced wine sensory properties such as structure, persistence, and mouth volume. Although the chemical parameter clearly indicated a higher polysaccharide release, this technique is not the most appropriate to evaluate fermentations involving the *Schizosaccharomyces* genus, as one of its peculiar characteristics is the possession of polysaccharides with a nature different from that of mannoproteins, i.e., galactomannoproteins [23,24]. This peculiarity should be taken into account in future studies regarding fermentation with the combination of *L. thermotolerans* and *S. pombe*.

### 2.12. Biogenic Amines

Nowadays, biogenic amines constitute the main food safety problem regarding food safety in wine [51]. Biogenic amines are mainly produced by LB during MLF [42] or during uncontrolled LB contamination throughout different winemaking processes. The risk of biogenic amines is higher in wines with high pH, where LB can easily develop and are more efficient in producing biogenic amines [51]. This problem is traditionally managed by employing commercially selected LB unable to produce biogenic amines such as histamine. Another common preventive measure is preventing undesirable wild LB contamination during AF from taking place using antibacterial additives such as sulfur dioxide or chitosan [51]. Other new control or preventive measures are based on avoiding MLF. *S. pombe* consumes all malic acid during AF, as well as all of the sugars, in a similar way to *S. cerevisiae*. This fact means that wines fermented by *S. pombe* do not require MLF stabilization; contamination by LB is not possible, as there are no potential subtracts to be metabolized by LB. Additionally, *L. thermotolerans* usually reduces pH by several decimal points, which also provides additional protection against LB. These are the arguments that indicate combined LT+SP use as an alternative to produce wines with low contents of biogenic amines. There are two studies that have analyzed biogenic amines in LT+SP fermentations [30,32], both of which report no statistical differences between combined LT+SP fermentations and the pure *S. cerevisiae* control. However, in the controls that performed MLF, there were slight increases in biogenic amines, such as histamine, by about 1 mg/L [30] or 0.44 mg/L [32], or tyramine, by about 0.11 mg/L [30] or 0.04 mg/L [32].

### 2.13. Ethyl Carbamate Precursors

Ethyl carbamate constitutes the third major food safety problem reported in wines after biogenic amines and ochratoxin A [51]. Several countries have stabilized the legal limits for this compound, which is considered carcinogenic. The main origin of ethyl carbamate in wine is the inevitable chemical combination between urea and ethanol, the reason of which is that urea is considered the main precursor of ethyl carbamate in winemaking. The second method of formation is during MLF by specific strains of LB. The classical way of managing this health problem from an industrial point of view was originally based on the use of commercial urease enzymes, which can completely remove urea. Another modern approximation consists of using of yeast genera in the initial starters that naturally possess urease activity. Until now, urease activity has been reported only for 18 yeast species that belong to seven non-*Saccharomyces* genera, among which *S. pombe* is included. The studies that report urea concentrations in final wines fermented with LT+SP combinations report urea values of about 0 mg/L, while the controls fermented with classical strategies show a few milligrams of urea per liter of wine [30,32,33,36].

### 2.14. Aroma Compounds

*S. pombe* is described as a lower producer of higher alcohols and esters than *S. cerevisiae,* varying from 25% to 66% [7,22]. On the other hand, *L. thermotolerans* is, on some occasions, described as a lower metabolizer of higher alcohols, while in other studies, is described as a higher producer than *S. cerevisiae* controls [19]. These discrepancies are explained due to the high strain variability, reported to reach 37% for *L. thermotolerans* [8,19,50,54,55,56]. Nevertheless, most studies report *L. thermotolerans* as a higher producer of esters than *S. cerevisiae* [19].

Selecting strains with low higher alcohol production is increasing in scientific interest, in order to produce high varietal wines such as those of grape varieties with high contents of terpenes or thiols [48], as these varietal aromas are occasionally masked by higher concentrations of higher alcohols that exceed 500 mg/L.

Combined LT+SP fermentations show the greatest statistical differences in the ethyl lactate volatile compound compared to the *S. cerevisiae* (SC) control. These differences are lower when compared to controls that perform MLF (SC+MLF); in these cases, the final differences depend on the initial malic acid metabolized to lactic acid by LB.

In the studies where a control performs MLF, combined LT+SP fermentations are reported to produce wines with lower concentrations of undesirable volatile compounds such as ethyl acetate or diacetyl [32].

The first study to report a volatile aroma compound analysis regarding combined LT+SP fermentations reported a lower production of higher alcohols than the *S. cerevisiae* control [32], as well as a lower production of 1-propanol (25%), isobutanol (20%), 2-methyl-butanol (30%), and 3-methyl-butanol (35%) than the regular *S. cerevisiae* control. The control used, which performed classical MLF, did not result in any statistically significant increases in higher alcohols compared to the regular *S. cerevisiae* control. Furthermore, the combined LT+SP fermentations resulted in higher levels of esters than the regular *S. cerevisiae* control due to the higher production of ethyl lactate, i.e., by 76%. The difference is explained by the lactic acid formation produced by *L. thermotolerans* metabolism. However, the control, which performed classical MLF, did not show differences in ethyl lactate from LT+SP but produced higher concentrations of ethyl acetate and diacetyl, i.e., by 39% and 80%, respectively.

Later studies reported combined LT+SP fermentations produced higher levels of 1-propanol and isobutanol, by about 50% compared to the SC control [37]. On the other hand, the *S. cerevisiae* control had higher concentrations of the main higher alcohols, such as 2-metyl-1-butanol, varying from 8% to 29%, and of 2,3-butanediol, varying from 15% to 85%, depending on the initial inoculum proportion between *S. pombe* and *L. thermotolerans* [37]. The total ester concentration was higher for combined LT+SP fermentations, varying from 20% to 39%, due to ethyl lactate production by *L. thermotolerans*. Although the study reported a higher production of diacetyl for LT+SP fermentations, by about 1 mg/L, no control that performed MLF was included. During that MLF process, the content of the volatile compound could have significantly increased before achieving final microbial stabilization.

A later study reported combined LT+SP fermentation produced higher concentrations of higher alcohols than the *S. cerevisiae* control, varying from 12% to 30% depending on the enrichment of catechin or procyanidin [34]. Regarding esters, the opposite effect was reported, as combined LT+SP fermentations resulted in higher final concentrations in fruity esters that varied from 63% to 69% depending on the enrichment of catechin or procyanidin. The final total volatile concentrations were always lower for combined LT+SP fermentation, varying from 29% to 41% depending on the catechin or procyanidin treatment.

The latest study reported combined LT+SP fermentation to produce fewer higher alcohols than the *S. cerevisiae* control, varying from 13% to 21% depending on the *L. thermotolerans* and *S. pombe* combination modality [39]. The *S. pombe* control showed a final higher alcohol concentration that was 50% lower than that of the *S. cerevisiae* control, which indicates that this phenomenon can be attributed to *S. pombe* metabolism. Nevertheless, combined LT+SP fermentations showed higher concentrations of specific higher alcohols such as 3-ethoxy-propanol, N-hexanol, N-butanol, 2-nonanol, and miristyl alcohol, which were not detected in the *S. cerevisiae* control.

The abovementioned study also showed combined LT+SP fermentation produced fewer esters than the *S. cerevisiae* control, varying from 38% to 49% depending on the *L. thermotolerans* and *S. pombe* combination modality [39]. Although the study analyzed 26 fermentative esters, ethyl lactate was not included. Combined LT+SP fermentation did not produce ethyl butyrate, but did produce specific esters that were not produced by *S. cerevisiae,* such as ethyl benzoate, ethyl phenyl acetate, heptyl formate, methyl salicylate, diethyl succinate, ethyl launate, caprylic acid-2-phenylethyl ester, ethyl trans-2-hexenoate, and methyl palmitate.

Furthermore, it was shown in the aforementioned study that the combined LT+SP fermentation produced fewer volatile acids than the *S. cerevisiae* control, varying from 46% to 49% depending on the *L. thermotolerans* and *S. pombe* combination modality [39]. Some of the fatty acids reported only for the combined LT+SP fermentation included oleic acid, geranic acid, and lauric acid; however, combined LT+SP fermentation did not result in some fatty acids produced by *S. cerevisiae* such as 2-methyl hexanoic acid and palmitic acid.

Combined LT+SP fermentation resulted in higher concentrations of terpenes, varying from 30% to 53%, compared with the *S. cerevisiae* control. Combined LT+SP fermentation resulted in terpenes not reported for *S. cerevisiae* control fermentation such as alpha-terpineol, linalool, citronellol, geraniol, trans-nerolidol, and geranylcetone. The *S. cerevisiae* control had a higher content of only nerolidol, while fol was only reported for *S. cerevisiae* fermentations. These results indicate that although combined LT+SP fermentations are commonly applied for red wine, it would be very interesting to apply the biotechnology to terpenic varieties, where it could increase the varietal terpenic aroma by up to 50%.

Other compounds that were only observed in combined LT+SP fermentations were decanoaldehyde and 2,6-di-tert-butyl-p-cresol.

The results of the study [39] show that the new biotechnology could produce other aroma compounds not reported before for conventional wines fermented by *Saccharomyces* and *O. oeni*. As only one study performed an MLF control, further studies must include an MLF control to verify the conclusions.

### 2.15. Color Intensity and Anthocyanins

LT+SP combined biotechnology, a part of the fast microbiological stabilization for red wines, also provides important advantages regarding the content of anthocyanins, which are the molecules responsible for the red wine color—one of the main quality parameters of red wine.

Fermentations involving sequential fermentations between *L. thermotolerans* and *S. cerevisiae* are commonly reported to increase color intensity in red wines by about 10% compared to the regular *S. cerevisiae* control [30]. This effect is mainly attributed to the decreases in pH of about 0.3–0.4 units because of the lactic acid formation produced during AF. This pH reduction increases the color intensity of anthocyanin molecules such as the flavylium ion [33]. Other studies report increases in total anthocyanin of 8% [20] or 14% [38] for sequential fermentation between *L. thermotolerans* and *S. cerevisiae,* which could be produced by lower anthocyanin absorption of the employed strains of *L. thermotolerans*. Nevertheless, the absorption ability is highly strain-dependent [19]. *S. pombe* usually produces wines with higher color intensity by about 10% than the *S. cerevisiae* controls that only perform AF [7] due to its ability to produce high concentrations of stable colored molecules related to pyruvic acid such as vitisin A [25,57]. Nevertheless, when *S. pombe* fermentations are compared to controls that perform MLF, the differences usually increase by up to 20% [7]. Several authors have reported color losses as one disadvantage of MLF related to LB glycosidase enzymes [58], as well as decreases in polymeric pigments [59], and a reduction of acetaldehyde that decreases vitisin B formation [60].

Reports of combined LT+SP fermentations always indicate higher color intensity than the *S. cerevisiae* control, for instance, by 7% [30], 12% [32], 9% [33], 40% [37], 5–16% [34], or 44% [39]. The studies that performed an MLF control reported that the effect always increased after the MLF, by 17% [30], 23% [32], or 26% [33]. Nevertheless, the *S. pombe* pure controls always showed higher color intensity than combined LT+SP fermentation, by about 7% [30], 10% [32], 9% [33], or 23% [39]. The improvements in color compared to the conventional fermentations are based on the combined effect of lower pH, which enhances the red color, the formation of stable color molecules such as vitisins, and avoiding the MLF process.

The first study regarding anthocyanin contents and combined LT+SP fermentations reported a higher content of total anthocyanins, of 9%, when compared to the regular *S. cerevisiae* control [33]. The difference increased up to 35% after MLF. The study also reported combined LT+SP fermentations resulted in higher concentrations of grape anthocyanins than the *S. cerevisiae* control that only performed AF, i.e., of delphinidin-3-oglucoside, cyanidin-3-O-glucoside, petunidin-3-O-glucoside, peonidin-3-O-glucoside, and malvidin-3-O-glucoside by 16%, 53%, 12%, 34%, and 2%, respectively. However, the differences increased up to 32%, 100%, 34%, 51%, and 30% compared to the control that performed MLF. LT+SP fermentation produced 17% more vitisin A and 43% more vitisin B than the *S. cerevisiae* control that only performed AF due to the high *S. pombe* pyruvic and acetaldehyde metabolism, which is related to the formation of these molecules. The differences increased up to 42% and 83% after MLF. LT+SP fermentation also showed higher levels of vitisin-A-acetylglucoside, cyanidin-3-O-(6″-0-acetylglucoside), petunidin-3-O-(6″-0-acetylglucoside), peonidin-3-O-(6″-0-acetylglucoside), and malvidin-3-O-(6″-0-acetylglucoside), with increases of 23%, 9%, 4%, 8%, and 7%, respectively, compared with the SC control that only performed AF. The differences increased up to 52%, 25%, 23%, 48%, and 38% after MLF. LT+SP fermentation resulted in higher final concentrations in cyanidin-3-O-(6″-0-p-coumaroylglucoside), petunidin-3-O-(6″-0-p-coumaroylglucoside), peonidin-3-O-(6″-0-p-coumaroylglucoside), and malvidin-3-O-(6″-0-p-coumaroylglucoside) than SC fermentation, i.e., of 10%, 18%, 37%, and 13%, and the differences increased up to 43%, 44%, 100%, and 40%, respectively, after MLF.

The results of the study [33] show that the main improvement in the anthocyanin content is due to the avoidance of MLF. Nevertheless, only one study regarding the LT+SP biotechnology included in the design of the experiment one control that performed MLF. Taking into account that almost every red wine available on the market is made by performing MLF, future studies should include an MLF control to verify the conclusions.

Another study reported an opposite effect for LT+SP fermentations that showed lower concentrations of malvidin-3-O-glucoside, by about 50%, compared to a control that performed regular AF by *S. cerevisiae* [34]. The content in vitisins varied from no statistical differences to 0.1 mg/L or more depending on the addition of catechin or procyanidin. LT+SP fermentations produced fewer oligomeric pigments, from 0.1 to 0.4 mg/L, depending on the catechin or procyanidin treatment. Nevertheless, the study reported, for all trials, higher final color intensities varying from 5% to 15% for LT+SP fermentations and from 6% to 9% for the total polyphenol index compared with the SC controls, depending on the different trials. Although it is not possible to study an MLF control, these differences could increase after LB action.

### 2.16. Sensory Evaluations

The studies that reported a sensory analysis for LT+SP fermentation reported better scores than SC controls, mainly for the acidity parameter due to the lactic acid metabolism of *L. thermotolerans*. Nevertheless, improvements in other sensory parameters such aroma quality and aroma intensity were also reported on some occasions [32,33]. Another study reported the combined fermentation (LT+SP) as the second best option after an SC strain control, but this option scored better than other SC strains used as another control [37]. Yet another study reported three different combined LT+SP fermentation trials that showed a higher fruity and flowery aroma than the SC control [39]. Although pure SP fermentations usually achieve higher scores in parameters such as color, structure, persistence, or mouth volume [33,36], LT+SP commonly obtains the highest overall impression due to a better balance of acidity, while SP fermentations are considered unbalanced due to their lack of acidity [32,33,36]. Studies that include an MLF control, on some occasions, report increases in negative sensory parameters such as acetic acid character and oxidation, as well as decreases in some positive parameters such as the color intensity [32,33,36]. Nevertheless, we must also take into account that these MLF processes take place in very alcoholic and high pH wines, where the risk of deviation is high.

## 3. Conclusions

New biotechnology based on the combined use of *L. thermotolerans* and *S. pombe* appears as a suitable alternative to ferment musts in warm viticulture areas, where performing malolactic fermentation at high pH levels with possible residual sugars and high ethanol concentrations may produce undesirable deviations in the quality of the wine.

Most studies report the combined use of *L. thermotolerans* and *S. pombe* to be faster than classical winemaking methodology that includes alcoholic fermentation and malolactic fermentation, improving the same microbial stability for the final product. *L. thermotolerans* and *S. pombe* fermentations are occasionally described to result in higher acidity, color intensity, and polysaccharide and fruity ester contents, with a lower pH, ethanol, higher alcohols, biogenic amines, and ethyl carbamate precursors.

The most common experimental design mistake regarding the combined use of *L. thermotolerans* and *S. pombe* is the absence of controls that perform malolactic fermentation by lactic bacteria. Half of the published studies included *S. cerevisiae* controls that only performed alcoholic fermentation. This point should be taken into account for future experimental designs to make it possible to compare similar stable wines from a microbiological point of view.

As the biotechnology based on the combined use of *L. thermotolerans* and *S. pombe* is new it requires future studies to optimize clonal selection, strain interactions and vinification conditions related to sulfur dioxide or fermentation temperature. As most studies are based on laboratory scale trials on pasteurized must, future studies also must perform fermentations at pilot scales or real industrial productions on fresh grape juice.

## Figures and Tables

**Table 1:** Main advantages and disadvantages of *Lachancea thermotolerans* and *Schizosaccharomyces pombe* in winemaking quality parameters.

*Lacchancea thermotolerans*		*Schizosaccharomyces pombe*	
Advantages	Disadvantages	Advantages	Disadvantages
Acidity ↑	Fermentative power ↓	Malic acid ↓	Acetic acid ↑
Lactic acid ↑	SO_2_ resistance ↓	Polysaccharides ↑	
Color intensity ↑		Color intensity ↑	
Acetic acid ↓		Color stability ↑	
Fruity esters ↑		Biogenic amines ↓	
		Ethyl carbamate ↓	

↑, higher activity; ↓, lower activity.

**Table 2:** Comparison of parameters in different studies involving fermentation combining the use of *Lachancea thermotolerans* and *Schizosaccharomyces pombe*.

		Fermentation Duration	Ethanol	Glycerol	Malic Acid	Lactic Acid	Citric Acid	Acetic Acid	pH	Total Acidity	Acetaldehyde	Polysaccharides	Biogenic Amines	Urea	Higher Alcohols	Esters	Terpenes	Color Intensity	Anthocyanins
Benito et al. 2015 [30]	SC	=	↑	=	=	=	=	=	=	n.d.a	n.d.a	n.d.a	=	=	n.d.a	n.d.a	n.d.a	=	n.d.a
	SC+MLF	↑↑↑	↑	=	↓↓	↑	↓	↑	↑	n.d.a	n.d.a	n.d.a	↑	n.d.a	n.d.a	n.d.a	n.d.a	↓↓	n.d.a
	LT+SC	↑	↓	↑	=	↑↑	=	↓	↓	n.d.a	n.d.a	n.d.a	=	=	n.d.a	n.d.a	n.d.a	↑	n.d.a
	LT+SC+MLF	↑↑↑	↓	↑	↓↓	↑↑↑	↓	↑	↑	n.d.a	n.d.a	n.d.a	↑	n.d.a	n.d.a	n.d.a	n.d.a	↓	n.d.a
	LT+SP	↑↑	↓	↑↑	↓↓	↑↑	=	=	↓	n.d.a	n.d.a	n.d.a	=	↓↓	n.d.a	n.d.a	n.d.a	↑↑	n.d.a
	SP	↑	↓	↑↑	↓↓	=	=	=	↑↑	n.d.a	n.d.a	n.d.a	=	↓↓	n.d.a	n.d.a	n.d.a	↑↑↑	n.d.a
Benito et al. 2016 [32]	SC	=	↑	=	=	=	=	=	=	n.d.a	n.d.a	n.d.a	=	=	↑	=	n.d.a	=	n.d.a
	SC+MLF	↑↑↑	↑	=	↓↓	↑	↓	↑	↑	n.d.a	n.d.a	=	↑	↑	↑	↑	n.d.a	↓↓	n.d.a
	LT+SC	↑	↓	↑	=	↑↑	=	=	↓	n.d.a	n.d.a	n.d.a	=	=	↓	↑↑	n.d.a	↑	n.d.a
	LT+SC+MLF	↑↑↑	↓	↑	↓↓	↑↑↑	↓	↑	↑	n.d.a	n.d.a	↓	↑	↑	↑	↑	n.d.a	↓	n.d.a
	LT+SP	↑↑	↓	↑↑	↓↓	↑↑	=	=	↓	n.d.a	n.d.a	↑	=	↓↓	↓	↑↑	n.d.a	↑↑	n.d.a
	SP	↑	↓	↑↑↑	↓↓	=	=	=	↑↑	n.d.a	n.d.a	↑↑	=	↓↓	↓↓	↓	n.d.a	↑↑↑	n.d.a
Benito et al. 2017 [33]	SC	=	↑	=	=	=	=	=	=	n.d.a	=	n.d.a	n.d.a	=	n.d.a	n.d.a	n.d.a	=	=
	SC+MLF	↑↑↑	↑	=	↓↓	↑	↓	↑	↑	n.d.a	↓↓	n.d.a	n.d.a	↑	n.d.a	n.d.a	n.d.a	↓↓	↓
	LT+SC	↑	↓	↑	=	↑↑	=	=	↓	n.d.a	↓	n.d.a	n.d.a	=	n.d.a	n.d.a	n.d.a	↑	↑
	LT+SC+MLF	↑↑↑	↓	↑	↓↓	↑↑↑	↓	↑	↑	n.d.a	↓↓	n.d.a	n.d.a	↑	n.d.a	n.d.a	n.d.a	↓	↓
	LT+SP	↑↑	↓	↑↑	↓↓	↑↑	=	=	↓	n.d.a	↑	n.d.a	n.d.a	↓↓	n.d.a	n.d.a	n.d.a	↑↑	↑
	SP	↑	↓	↑↑	↓↓	=	=	=	↑↑	n.d.a	↑↑	n.d.a	n.d.a	↓↓	n.d.a	n.d.a	n.d.a	↑↑↑	↑↑
Fresno et al. 2017 [37]	SC	=	↑	=	=	=	n.d.a	↓	=	=	n.d.a	n.d.a	n.d.a	n.d.a	↑	=	n.d.a	=	↑
	SC+MLF	n.d.a	n.d.a	n.d.a	n.d.a	n.d.a	n.d.a	n.d.a	n.d.a	n.d.a	n.d.a	n.d.a	n.d.a	n.d.a	n.d.a	n.d.a	n.d.a	n.d.a	n.d.a
	LT+SC	=	↓	↑	=	↑↑	n.d.a	↓	=	↓	n.d.a	n.d.a	n.d.a	n.d.a	↑	=	n.d.a	=	=
	LT+SC+MLF	n.d.a	n.d.a	n.d.a	n.d.a	n.d.a	n.d.a	n.d.a	n.d.a	n.d.a	n.d.a	n.d.a	n.d.a	n.d.a	n.d.a	n.d.a	n.d.a	n.d.a	n.d.a
	LT+SP	=	↓↓	↓	↓↓	↑	n.d.a	↑	↑	↓↓	n.d.a	n.d.a	n.d.a	n.d.a	↓	↑↑	n.d.a	↑	=
	SP	=	↑	↑↑	↓↓	=	n.d.a	↑↑	↑	↓↓	n.d.a	n.d.a	n.d.a	n.d.a	↓↓	↓	n.d.a	↑	↑↑
Escott et al. 2018a [34]	SC	=	↑	n.d.a	=	=	n.d.a	=	=	=	n.d.a	n.d.a	n.d.a	n.d.a	=	=	n.d.a	=	↑
	SC+MLF	n.d.a	n.d.a	n.d.a	n.d.a	n.d.a	n.d.a	n.d.a	n.d.a	n.d.a	n.d.a	n.d.a	n.d.a	n.d.a	n.d.a	n.d.a	n.d.a	n.d.a	n.d.a
	LT+SC	=	↑	n.d.a	↓	↑	n.d.a	↓↓	=	↓	n.d.a	n.d.a	n.d.a	n.d.a	↑↑	↑	n.d.a	↑	=
	LT+SC+MLF	n.d.a	n.d.a	n.d.a	n.d.a	n.d.a	n.d.a	n.d.a	n.d.a	n.d.a	n.d.a	n.d.a	n.d.a	n.d.a	n.d.a	n.d.a	n.d.a	n.d.a	n.d.a
	LT+SP	=	↓	n.d.a	↓↓	↑	n.d.a	↓↓	↑	↓↓	n.d.a	n.d.a	n.d.a	n.d.a	↑	↑↑	n.d.a	↑	=
	SP	=	n.d.a	n.d.a	n.d.a	n.d.a	n.d.a	n.d.a	n.d.a	n.d.a	n.d.a	n.d.a	n.d.a	n.d.a	n.d.a	n.d.a	n.d.a	n.d.a	n.d.a
Escott et al. 2018b [35]	SC	n.d.a	n.d.a	n.d.a	n.d.a	n.d.a	n.d.a	n.d.a	n.d.a	n.d.a	n.d.a	n.d.a	n.d.a	n.d.a	n.d.a	n.d.a	n.d.a	n.d.a	n.d.a
	SC+MLF	n.d.a	n.d.a	n.d.a	n.d.a	n.d.a	n.d.a	n.d.a	n.d.a	n.d.a	n.d.a	n.d.a	n.d.a	n.d.a	n.d.a	n.d.a	n.d.a	n.d.a	n.d.a
	LT+SC	=	n.d.a	n.d.a	n.d.a	n.d.a	n.d.a	n.d.a	n.d.a	n.d.a	n.d.a	n.d.a	n.d.a	n.d.a	=	=	n.d.a	=	=
	LT+SC+MLF	n.d.a	n.d.a	n.d.a	n.d.a	n.d.a	n.d.a	n.d.a	n.d.a	n.d.a	n.d.a	n.d.a	n.d.a	n.d.a	n.d.a	n.d.a	n.d.a	n.d.a	n.d.a
	LT+SP	=	n.d.a	n.d.a	n.d.a	n.d.a	n.d.a	n.d.a	n.d.a	n.d.a	n.d.a	n.d.a	n.d.a	n.d.a	=	=	n.d.a	↑	=
	SP	n.d.a	n.d.a	n.d.a	n.d.a	n.d.a	n.d.a	n.d.a	n.d.a	n.d.a	n.d.a	n.d.a	n.d.a	n.d.a	n.d.a	n.d.a	n.d.a	n.d.a	n.d.a
Benito et al. 2019 [36]	SC	=	↑	=	=	=	=	=	=	n.d.a	=	=	n.d.a	=	n.d.a	n.d.a	n.d.a	=	n.d.a
	SC+MLF	↑↑↑	↑	=	↓↓	↑	↓	↑	↑	n.d.a	↓↓	=	n.d.a	↑	n.d.a	n.d.a	n.d.a	↓↓	n.d.a
	LT+SC	↑	↓	=	=	↑↑	=	=	↓	n.d.a	↓	↓	n.d.a	=	n.d.a	n.d.a	n.d.a	↑	n.d.a
	LT+SC+MLF	↑↑↑	↓	=	↓↓	↑↑↑	↓	↑	↑	n.d.a	↓↓	↓	n.d.a	↑	n.d.a	n.d.a	n.d.a	↓	n.d.a
	LT+SP	↑↑	↓	↑	↓↓	↑↑	=	=	↓	n.d.a	↑	↑	n.d.a	↓↓	n.d.a	n.d.a	n.d.a	↑↑	n.d.a
	SP	↑	↓	↑	↓↓	=	=	=	↑↑	n.d.a	↑↑	↑↑	n.d.a	↓↓	n.d.a	n.d.a	n.d.a	↑↑↑	n.d.a
Wang et al. 2019 [39]	SC	=	↑	n.d.a	=	↑	n.d.a	=	=	=	n.d.a	n.d.a	n.d.a	n.d.a	↑	↑	=	=	n.d.a
	SC+MLF	n.d.a	n.d.a	n.d.a	n.d.a	n.d.a	n.d.a	n.d.a	n.d.a	n.d.a	n.d.a	n.d.a	n.d.a	n.d.a	n.d.a	n.d.a	n.d.a	n.d.a	n.d.a
	LT+SC	n.d.a	n.d.a	n.d.a	n.d.a	n.d.a	n.d.a	n.d.a	n.d.a	n.d.a	n.d.a	n.d.a	n.d.a	n.d.a	n.d.a	n.d.a	n.d.a	n.d.a	n.d.a
	LT+SC+MLF	n.d.a	n.d.a	n.d.a	n.d.a	n.d.a	n.d.a	n.d.a	n.d.a	n.d.a	n.d.a	n.d.a	n.d.a	n.d.a	n.d.a	n.d.a	n.d.a	n.d.a	n.d.a
	LT+SP	↑↑	↓↓	n.d.a	↓	↑↑	n.d.a	↓	↓	↑	n.d.a	n.d.a	n.d.a	n.d.a	↓	=	↑↑	↑	n.d.a
	SP	↑↑	↓	n.d.a	↓	=	n.d.a	↓↓	↑	↓	n.d.a	n.d.a	n.d.a	n.d.a	↓↓	=	↑↑	↑↑	n.d.a

↑, higher activity; ↓, lower activity; =, similar activity; n.d.a, no data available; SC, *S. cerevisiae* pure alcoholic fermentation; SC+MLF, alcoholic fermentation by *S. cerevisiae* and malolactic fermentation by *O. oeni*; LT+SC, alcoholic fermentation combining *L. thermotolerans* and *S. cerevisiae*; LT+SC+MLF, alcoholic fermentation combining *L. thermotolerans* and *S. cerevisiae* and malolactic fermentation by *O. oeni*; LT+SP, alcoholic fermentation combining *L. thermotolerans* and *S. pombe;* SP, *S. pombe* pure alcoholic fermentation.

## Abbreviations

LT	*Lachancea thermotolerans*
SP	*Schizosaccharomyces pombe*
LT+SP	*L. thermotolerans* and *S. pombe* combined alcoholic fermentation
SC	*Saccharomyces cerevisiae*
MLF	Malolactic fermentation
AF	Alcoholic fermentation
SC+MLF	Alcoholic fermentation by *S. cerevisiae* followed by malolactic fermentation by *O. oeni*.
LB	Lactic bacteria
